# Yap/Taz activity is associated with increased expression of phosphoglycerate dehydrogenase that supports myoblast proliferation

**DOI:** 10.1007/s00441-023-03851-w

**Published:** 2024-01-06

**Authors:** Marius Meinhold, Sander Verbrugge, Andi Shi, Martin Schönfelder, Lore Becker, Richard T. Jaspers, Peter S. Zammit, Henning Wackerhage

**Affiliations:** 1 School of Medicine and Health, Technical University of Munich, Connollystrasse 32, 80809 Munich, Germany; 2 Laboratory for Myology, Department of Human Movement Sciences, Faculty of Behavioural and Movement Sciences, Amsterdam Movement Sciences, Vrije Universiteit Amsterdam, De Boelelaan 1108, 1081 HZ Amsterdam, The Netherlands; 3 Department of Prosthodontics, Guangdong Engineering Research Center of Oral Restoration and Reconstruction, Guangzhou Key Laboratory of Basic and Applied Research of Oral Regenerative Medicine, Affiliated Stomatology Hospital of Guangzhou Medical University, Guangzhou, China; 4 Randall Centre for Cell and Molecular Biophysics, King’s College London, New Hunt’s House, Guy’s Campus, London, SE1 1UL UK; 5 Institute of Experimental Genetics, Helmholtz Zentrum München, German Research Center for Environmental Health, German Mouse Clinic, Ingolstädter Landstrasse 1, 85764 Neuherberg, Germany

**Keywords:** Yap, Taz, Phosphoglycerate dehydrogenase, Phgdh, Skeletal muscle, Myoblast

## Abstract

In skeletal muscle, the Hippo effector Yap promotes satellite cell, myoblast, and rhabdomyoblast proliferation but prevents myogenic differentiation into multinucleated muscle fibres. We previously noted that Yap drives expression of the first enzyme of the serine biosynthesis pathway, phosphoglycerate dehydrogenase (*Phgdh*). Here, we examined the regulation and function of Phgdh in satellite cells and myoblasts and found that Phgdh protein increased during satellite cell activation. Analysis of published data reveal that *Phgdh* mRNA in mouse tibialis anterior muscle was highly expressed at day 3 of regeneration after cardiotoxin injection, when markers of proliferation are also robustly expressed and in the first week of synergist-ablated muscle. Finally, siRNA-mediated knockdown of PHGDH significantly reduced myoblast numbers and the proliferation rate. Collectively, our data suggest that Phgdh is a proliferation-enhancing metabolic enzyme that is induced when quiescent satellite cells become activated.

**Supplementary Information:**

The online version contains supplementary material available at 10.1007/s00441-023-03851-w.

## Introduction

The evolutionary conserved Hippo signal transduction network comprises the Hippo kinase cascade and other signalling proteins that regulate activity and localisation of the transcriptional co-factors Yap (gene *Yap1*) and Taz (gene *Wwtr1*) (Ma et al. 2019). Unphosphorylated, nuclear, and active Yap and Taz bind to Tead1-4 transcription factors that bind to CATTCC/GGAATG binding elements to regulate expression of target genes such as Ctgf (Zhao et al. 2008).

A major function of active Yap/Taz-Tead1-4 complexes is to stimulate cell proliferation, e.g. during development, stem cell-mediated regeneration, organ growth, and in cancer. This was demonstrated in vivo in flies (Huang et al. 2005) and mouse liver (Camargo et al. 2007; Dong et al. 2007) and partially explains the link between Yap abundance or activity and cancer (Kulkarni et al. 2020; Moroishi et al. 2015; Zanconato et al. 2016).

Skeletal muscle is able to grow, repair, and regenerate due to resident muscle stem cells called satellite cells that activate and proliferate to generate myoblast progeny (Forcina et al. 2019; Zammit 2017). The Hippo effector Yap also regulates proliferation of muscle cells (Wackerhage et al. 2014). Specifically, we showed that Yap regulates the proliferation but inhibits differentiation of murine C2C12 myoblasts (Watt et al. 2010) and primary satellite cells (Judson et al. 2012). Moreover, persistent hyperactivity of Yap in activated satellite cells is sufficient to cause embryonal rhabdomyosarcomas to develop in mice. Such rhabdomyosarcomas are a ‘myoblast’ cancer, and many human embryonal rhabdomyosarcomas express high levels of YAP (Tremblay et al. 2014).

The rate of proliferation is primarily regulated by cell cycle-regulating signalling proteins such as cyclins and cyclin-dependent kinases (Cdk's) (Nurse 2000) which are also regulated by Hippo proteins. For example, we found that the overexpression of YAP1 S127A in myoblasts increased expression of cell cycle regulators *Cdk1*, *Cdk6*, and *cyclins A2*, *B1*, *E1*, and *E2* as well as expression of the proliferation marker *Mki67* (Judson et al*. *2012).

One proliferation-associated mechanism is accretion of biomass, as a proliferating cell needs to increase its biomass to generate two daughter cells. This requires metabolic reprogramming, which has especially been studied in cancer. Warburg discovered the first link between metabolism and proliferation when he reported that cancer cells take up more glucose and synthesize more lactate than healthy, less-proliferating cells (Warburg 1925; Warburg et al. 1927). After many decades, it became clear that the metabolic reprogramming of cancer and normal proliferating cells serves several roles. One key function is channelling substrates from energy metabolism pathways into anabolic pathways such as nucleotide, amino acid, and phospholipid synthesis (DeBerardinis and Chandel 2020; Liberti and Locasale 2016; Vander Heiden and DeBerardinis 2017). Such metabolic reprogramming occurs in proliferating muscle cells (Fu et al. 2015; Ryall 2013), cardiomyocytes (Honkoop et al. 2019) and differentiated muscle cells (Stadhouders et al. 2023; Wackerhage et al. 2022). Moreover, we found that the cancer-associated pyruvate kinase muscle 2 (Pkm2) is a regulator of myotube size (Verbrugge et al. 2020).

Based on our finding that Yap regulates myoblast proliferation and cell cycle-regulating proteins (Judson et al*. *2012; Tremblay et al*. *2014; Watt et al*. *2010), we searched gene expression datasets for metabolic reprogramming–associated genes regulated by high Yap activity. Overexpression of YAP1 S127A in mouse myoblasts increased expression of all three enzymes of the serine biosynthesis pathway: phosphoglycerate dehydrogenase (*Phgdh*, EC 1.1.1.95), phosphoserine aminotransferase (*Psat1*, EC 2.6.1.52), and phosphoserine phosphatase (*Psph*, EC 3.1.3.3). This is interesting because Phgdh is involved in metabolic reprogramming in cancer (Locasale et al. 2011; Possemato et al. 2011). Even though serine is a non-essential amino acid, loss of *Phgdh* is embryonal lethal (Yoshida et al. 2004), suggesting that Phgdh has more functions than just synthesis of an amino acid that is abundant in a normal diet.

 The aim of this study was therefore to study the regulation and function of Phgdh in myoblasts. We reveal that Yap and Taz induce *Phgdh* expression in myoblasts, that Phgdh is highly expressed in situations where Yap activity is high, and that knockdown of Phgdh reduces proliferation.

## Methods

### Bioinformatical analyses

To examine whether *Phgdh*, *Psat1*, and *Psph* expression is higher in muscle cells with constitutively active YAP, we reanalysed microarray results of YAP S127A-expressing primary myoblasts from Judson et al. (Judson et al*. *2012). For tissue expression, we reanalysed a microarray dataset (GSE47198, Gene Expression Omnibus) from YAP S127A-driven rhabdomyosarcoma (Tremblay et al*. *2014).

To determine expression of PHGDH in skeletal muscle compared to other tissues, we retrieved expression figures from the Human Protein Atlas (https://www.proteinatlas.org/; (Uhlen et al. 2015)). Expression data for all organs can be found in the supplementary file (Supplementary Table S1).

To assess differences in developmental regulation of *Phgdh* between muscle allotypes, we retrieved the microarray dataset GSE903 from Cheng et al. (2004) and plotted the temporal profile of *Phgdh* expression of extraocular muscles and pooled gastrocnemius and soleus muscles (Cheng et al. 2004).

To compare abundance of PHGDH between a cultured muscle cell line (C2C12) and skeletal muscle tissue, we retrieved the supplemental datasets from Deshmukh et al. (Deshmukh et al. 2015).

To find out whether *Phgdh* expression differs between younger (5 days to 19 years) and older people (71 to 84 years), we retrieved the microarray dataset GSE4667 (Kang et al. 2005). Further, we examined whether the fibre-type-specific expression of PHGDH differs between younger and older adults by reanalysing the supplemental datasets from Murgia et al. (Murgia et al. 2017) and plotting the normalised protein abundance of PHGDH in type I and type IIa fibres of younger (22–27 years) and older adults (65–75 years).

To determine if regenerating skeletal muscle tissue increases expression of *Phgdh*, we retrieved the microarray dataset of cardiotoxin-injured TA muscles in Lukjanenko et al. (Lukjanenko et al. 2013).

Dystrophic muscles are highly abundant in proliferating myoblasts; therefore, we retrieved the supplementary data from Chemello et al. (Chemello et al. 2020) to reveal if deletion of Exon 51 of the *DMD* gene increases expression of *Phgdh*.

To examine whether *Phgdh* expression increases during overload-induced muscle hypertrophy, we retrieved the microarray dataset GSE47098 (Chaillou et al. 2013). We calculated expression relative to controls (day 0) during a time course of 14 days after synergist ablation.

Since hypoxia induces the nuclear translocation of YAP in various cell lines, we also retrieved a dataset of hypoxia-treated mouse plantaris muscles (GSE81286 from (Gan et al. 2017)).

### Muscle fibre isolation

Satellite cells and floating myofibres were isolated as described in elsewhere (Moyle and Zammit 2014). Mice aged between 8 and 12 weeks were killed by cervical dislocation and the extensor digitorum longus (EDL) muscles were isolated and digested as previously prescribed. Isolated myofibres were plated on Matrigel and the satellite cell–derived myoblasts then expanded using DMEM GlutaMAX (Invitrogen), with 30% fetal bovine serum (Gibco), 10% horse serum (Invitrogen Life Technologies), 1% chick embryo extract (MP), 10 ng/ml bFGF (PeproTech), and 1% penicillin/streptomycin (Sigma).

### Primary cell culture

Satellite cells were cultured in 6-well plates in proliferation medium or switched to differentiation medium for the stated duration as described elsewhere (Moyle and Zammit 2014).

### Immunolabelling

Isolated myofibres with their attached satellite cells were fixed with 4% PFA and washed 3 × for 5 min in PBS. Samples were permeabilized for 5 min with PBS containing 0.5% Triton X-100 and blocked for 1 h in 10% goat serum (ab7471; Abcam). Following blocking, isolated muscle fibres with associated satellite cells were incubated at 4 °C overnight with primary antibodies in PBS. We used the following antibodies: rabbit anti-Phgdh (ThermoFisher, PA5-27,578), mouse anti-Pax7 (DSHB), and mouse anti-MyoD (Dako). After washing with PBS (3 × 5 min), samples were incubated with secondary antibodies (ThermoFisher Scientific) in 10% goat serum for 1 h and again washed 3 × 5 min in PBS. Finally, samples were incubated with DAPI (1:1000, PBS) for 15 min after which a final wash of 5 min followed. Myofibres with associated satellite cells were mounted on glass slides with mounting medium (cat50001; ibidi) and a cover slip. Nail varnish was used to seal the cover slip to prevent dehydration (Moyle and Zammit 2014).

### RNA isolation

RNA was extracted from primary myogenic cells isolated from EDL of C57BL/10 mice using the RNeasy mini kit (QIAGEN, Cat#74,104). The extract was then centrifuged using a 5415R centrifuge (Eppendorf), at 10,000 rpm. Three hundred fifty microliters of buffer RLT was added; cells were then homogenized by passing the lysate through a blunt 19-gauge needle. Three hundred fifty microliters of 70% ethanol was added before centrifuging for 15 s. Seven hundred microliters of buffer RW1 was added to cells and centrifuged for 15 s. Five hundred microliters of buffer RPE was added and the cells centrifuged for 30 s. A further 500 μl of RPE was added and cells were centrifuged for 2 min. New collection tubes were added to each spin column and cells were centrifuged at maximum speed (13,200 rpm) for 1 min. Forty microliters of RNase-free water was added (dropwise) and centrifuged for 1 min to provide an eluted RNA pellet.

Reverse transcription was carried out using the QuantiTect kit (QIAGEN, Cat#205,311). Optical density analysis using a NanoDrop ND-1000 spectrophotometer (Labtech) quantified RNA concentration. RNA was made up to 12 μl using RNase-free water. Two microliters of DNA wipeout buffer was added and samples were placed in a water bath for 2 min at 42 °C. Samples were then placed on ice for 2 min. One microliter of primer mix was added and samples were placed in a heat block for 2 min at 70 °C, followed by an additional 2 min on ice. Four microliters of RT buffer and 1 μl reverse transcriptase enzyme were added to each sample and placed in a water bath for 30 min at 42 °C. Samples were placed in a heat block for 3 min at 95 °C. Finally, the cDNA concentration was measured using the NanoDrop.

### RT-qPCR

Real-time quantitative PCR (qPCR) was carried out using the Mx3000P qPCR system (Agilent Technologies) using Mesa Blue Master Mix (MM) solution according to manufacturer’s instructions (Eurogentec Cat#05- SY2X-03 + WOUB). Primer sequences are given in Table 1. The qPCR cycle consisted of a 10-min incubation at 95 °C for qPCR enzyme activation, followed by 40 cycles at 95 °C for 30 s, a qPCR amplification period of 30 s at 60 °C, and a polymerase extension period of 30 s at 72 °C. Data was normalised against *Tbp*.

**Table 1:** Primers for qPCR

**Target gene**	**Primer sequences (5′-3′)**
*Phgdh*	TGGTGAACGCTAAGCTACTGG CAGGGCCACAGTCAGGAG
*Psat1*	AGAATCTTGTGAGGGAATTGCT TTTAAGGGGACAGCACTGAAC
*Psph*	CGTTGCTGCAAAGCTCAATA CTGTCGGCTGCATCTCATC
*Pax7*	CCGTGTTTCTCATGGTTGTG GAGCACTCGGCTAATCGAAC
*Myog*	CTACAGGCCTTGCTCAGCTC AGATTGTGGGCGTCTGTAGG
*Myh1*	GTCCAAAGCCAACAGTGAAG CTTCTGTTTCCATTCTGCCA
*Tbp*	ATCCCAAGCGATTTGCTG CCTGTGCACACCATTTTTCC

### siRNA transfection

*Phgdh* in C2C12 cells was knocked down using silencer RNA (siPHGDH). C2C12 myoblasts were cultured as previously described (Hillege et al. 2020). Once they reached 30% confluence, cells were transfected with siRNA targeted against *Phgdh* (Table 2) using the liposome-mediated method Lipofectamine™ RNAiMAX Reagent (Invitrogen, 13,778–030). As a negative control, a non-targeting silence RNA sequence (siControl, Table2) was used (Silencer® Select Negative Control #1 siRNA, Invitrogen 4,390,843). siRNA was diluted in Opti-MEM medium (Opti-MEM® I Reduced-Serum Medium, 31,985,070) and incubated for 5 min with Lipofectamine mixture. RNA-Lipofectamine complexes with a final concentration of 20 nM were added to each well.

**Table 2:** siRNA sequences

**Target gene**	**Sequences (5′-3′)**
siPhgdh	CCCGAAUGCAAUCCUUUGGTT CCATCCAATCGGTAGTAGTAGCG
siControl	AGUACUGCUUACGAUACGGTT CCGUAUCGUAAGCAGUACUTT

### EdU assay

After 24 h and 48 h of siRINA transfection, EdU (5-ethynyl2′-deoxyuridine) was incubated for 2 h with a final concentration of 10 µM. EdU staining was performed using the Alexa Fluor 647 with the Click-iT EdU Imaging Kit (ThermoFisher, C10640) according to the manufacturer’s instruction. Cell nuclei were visualized by Hoechst 33,342 staining. The percentage of EdU-positive cells was determined by dividing the EdU-positive cells by the total number of nuclei per field (five images in each well) using ImageJ software.

### RNA isolation for siRNA transfection experiments

After washing C2C12 cells with PBS, cells were lysed in TRI reagent (Invitrogen Solution, 1,131,129,240). Two hundred nanogram RNA was isolated using RiboPure kit (ThermoFisher Scientific, AM1924) and converted to cDNA with high-capacity RNA to cDNA master mix (Invitrogen, 12,023,679) with 2720 thermocycler (Applied Biosystems). cDNA was 10 × diluted and stored at –20 °C.

### Real-time qPCR for siRNA transfection experiments

cDNA samples were analysed in duplicate using real-time qPCR with a fluorescent SYBR Green Master Mix (Fisher Scientific, Cat#10,556,555) (see for details Stadhouders et al. 2023). Target gene expression was normalised to 18S as housekeeping gene (primers in Table 3).

**Table 3:** Primers for qPCR for siRNA transfection experiments

**Target gene**	**Primer sequences (5′-3′)**
*Phgdh*	CCCACTATGATTGGCCTCCT AGACACCATGGAGGTTTGGT
*18S*	GTAACCCGTTGAACCCCATT CCATCCAATCGGTAGTAGCG

### Statistics

Relative expression levels between proliferating and differentiated primary muscle stem cells were measured in three replicates (three different mice), and significance was tested using a two-tailed Student *t* test in Microsoft Excel. Significance was set at *p* < 0.05.

#### For siRNA experiments

Before analysing the data, the percentage of EdU-positive cells was determined. The fold change was calculated by dividing the percentage of EdU-positive cells of silencing PHGDH by the average percentage of EdU-positive cells of the control group. Normal distribution was tested with Shapiro–Wilk tests and equal variances were tested with Levene’s test. The effects of knockdown of *Phgdh* on gene expression and on total number of cells were determined using two-way analysis of variance (ANOVA). In the case of a significant ANOVA effect, a post hoc Bonferroni test was performed to determine significant difference between conditions. Significance was set at *p* < 0.05.

## Results

To identify genes whose expression is induced by active Yap which drives proliferation in murine C2C12 myoblasts (Watt et al*. *2010) and primary satellite cells (Judson et al*. *2012), we systematically reanalysed transcriptomic datasets of YAP1 S127A overexpression in mouse myoblasts and rhabdomyosarcomas induced by YAP1 S127A overexpression in activated satellite cells. This suggested that YAP1 S127A caused increased expression of *Phgdh* and *Psat1* which are the first two enzymes of the serine biosynthesis pathway (Table 4).

**Table 4:** Effect of YAP1 S127A overexpression in myoblasts on the expression of *Phgdh*, *Psat1*, and *Psph*

**Study and experiment**	***Phgdh***	***Psat1***	***Psph***
YAP1 S127A induction by doxycycline in mouse myoblasts (Judson et al*.* 2012)^a^	1.48↑ after 20 h 1.51↑ after 40 h	1.50↑ after 20 h 1.68↑ after 40 h	1.36↑ after 40 h
YAP1 S127A rhabdomyosarcoma versus skeletal muscle (Tremblay et al*.* 2014)	5.3↑	14.3↑	0.0

^a^Note that activated myoblasts already have high levels of Yap activity which may explain the relatively small expression changes (Judson et al*.* 2012)

Moreover, we previously reported that overexpression of constitutively active TAZ S89A similarly increased expression of *Phgdh*, *Psat1*, and *Psph* in C2C12 myoblasts (Mohamed et al. 2016). Collectively, this identifies the serine biosynthesis pathway as a target of the Hippo effectors Yap and Taz in myoblasts.

**Fig. 1:**
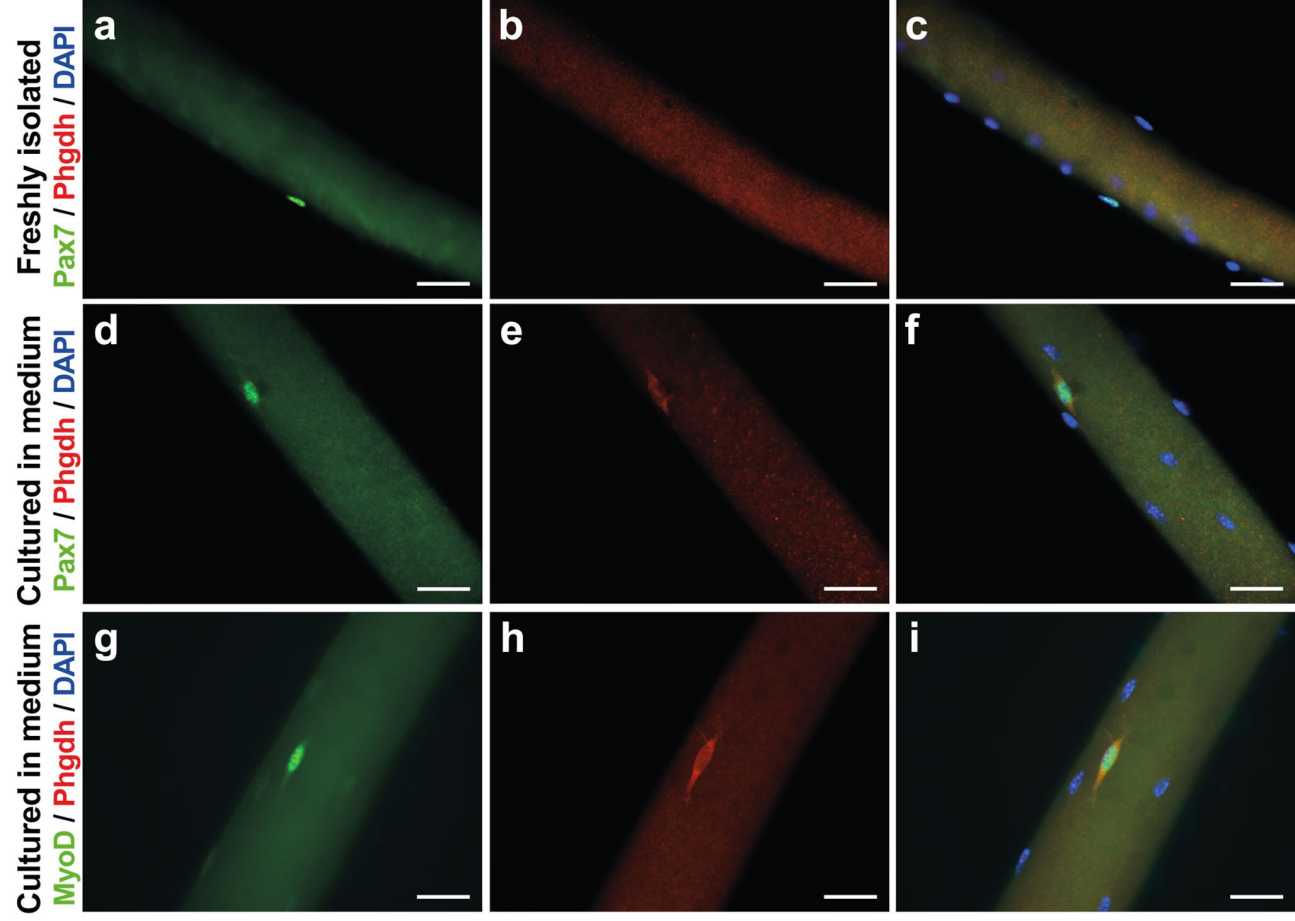
PHGDH abundance in satellite cells on freshly isolated myofibres. **a**–**c** Isolated EDL myofibres with their associated satellite cells were either immediately fixed or **d-i** cultured in proliferation medium for 24 h followed by fixation and immunolabelling for PAX7 (**a**, **c**, **d**, **f**), PHGDH (**b**, **c**, **e**, **f**, **h**, **i**), or MYOD (**g**, **i**). Scale bar represents 10 µm

When satellite cells become activated, Yap protein becomes detectable (Judson et al*. *2012). To investigate whether Phgdh protein similarly increases when satellite cells become activated and proliferate, we immunolabelled Phgdh in quiescent (Pax7+, MyoD−) and activated (Pax7+, MyoD+) satellite cells in their niche on isolated muscle fibres (Fig. 1). This showed that Phgdh protein was low in freshly isolated satellite cells that are positive for Pax7 (Fig. 1a–c). Phgdh protein then became visible in activated, MyoD+, and Pax7 + satellite cells when cultured in proliferation medium for 24 h (Fig. 1d–i). This suggests that Phgdh protein becomes abundant, like Yap (Judson et al*. *2012), in activated satellite cells.

**Fig. 2:**
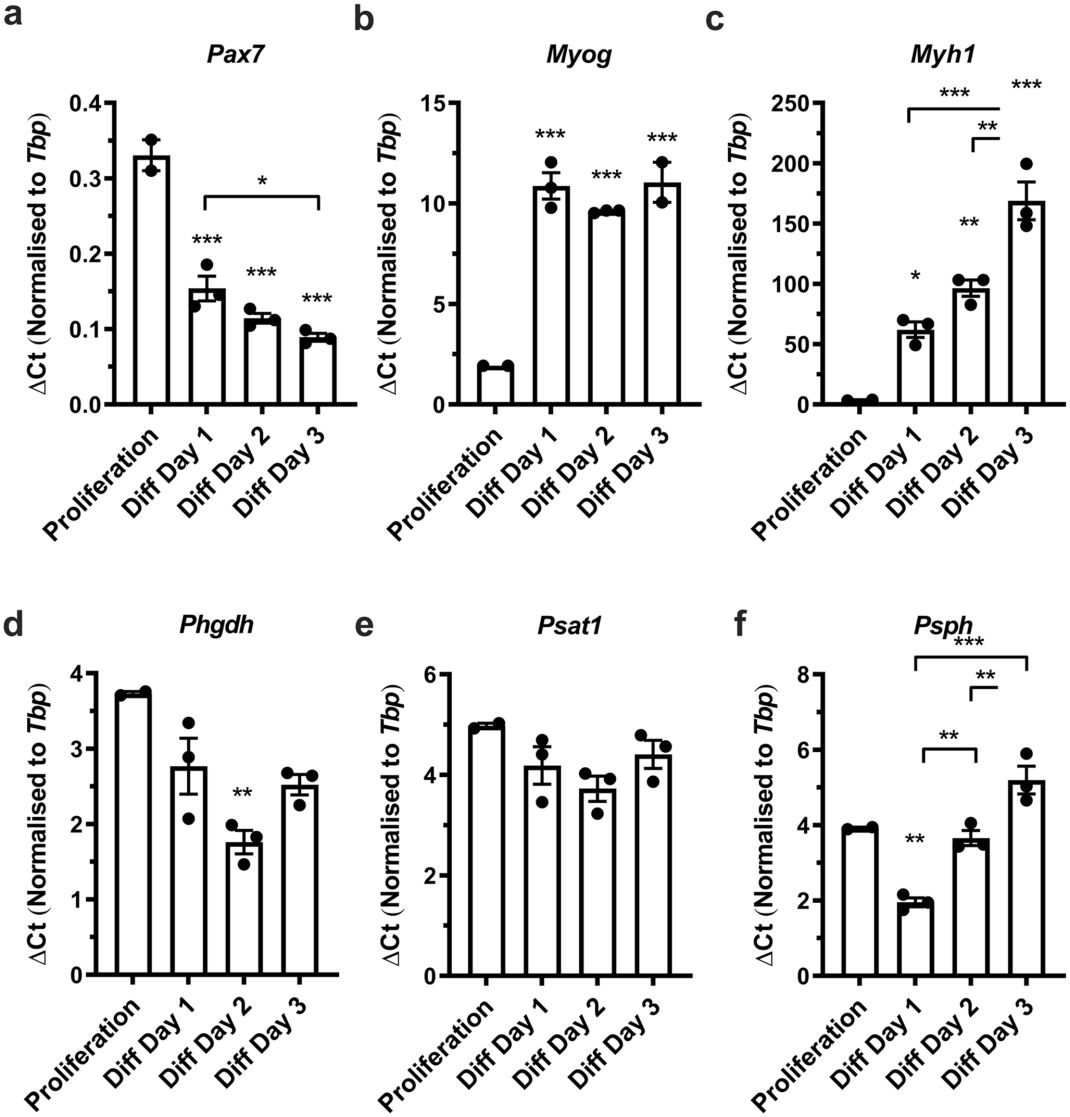
Gene expression in proliferating and differentiating primary satellite cells. **a-f** mRNA expression during proliferation, and 1st, 2nd, and 3rd day of differentiation (Diff) for **a** *Pax7*; **b** *Myog*; **c** *Myh1*; **d** *Phgdh*; **e** *Psat1*; and **f** *Psph* (*n* = 2–3). An asterisk denotes significantly different from proliferation or between indicated conditions (*p* < 0.05), two asterisks denote a *p* < 0.01, and three asterisks *p* < 0.001

Next, we analysed expression of *Phgdh*, *Psat1*, and *Psph* during myoblast differentiation into multinucleated myotubes (Fig. 2). Decreased expression of *Pax7* (Fig. 2a), combined with significant increases in *Myog* (Fig. 2b) and *Myh1* (Fig. 2c), confirmed myogenic differentiation. During differentiation, *Phgdh* (Fig. 2d) and *Psph* (Fig. 2f) transiently declined but then increased again whereas *Psat1* (Fig. 2e) remained relatively unchanged.

**Fig. 3:**
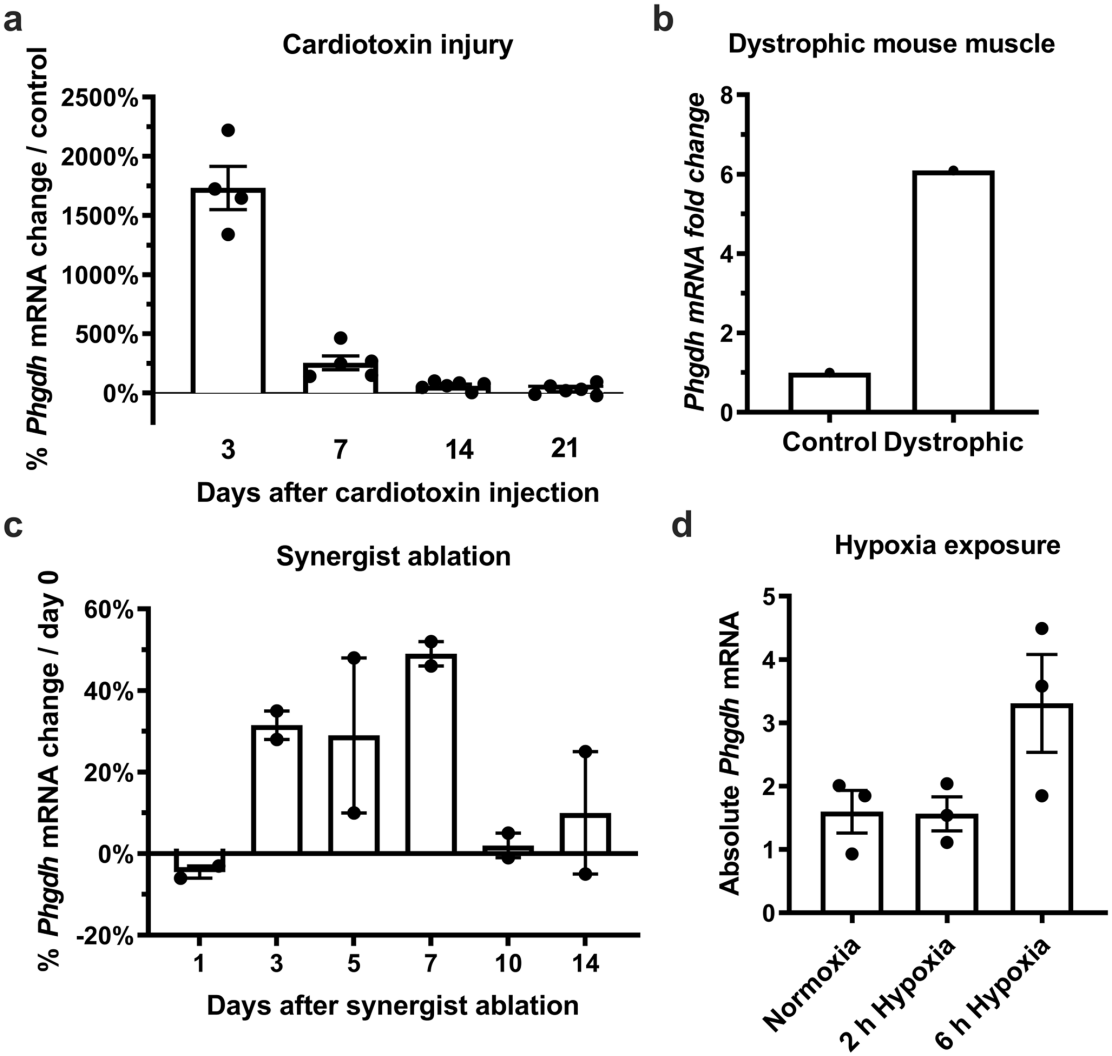
Reanalysed datasets of *Phgdh* expression show changes in expression during muscle perturbation in mice. **a** Cardiotoxin-induced injury increases *Phgdh* expression 3 and 7 days post-injury in mouse tibialis anterior (Lukjanenko et al*. *2013); **b** *Phgdh* expression is higher in TA muscle of dystrophic mice (*n* = 3, individual data not available; (Chemello et al*. *2020);** c** bilateral synergist ablation increases *Phgdh* expression 3, 5, and 7 days in mouse plantaris muscle (data points reflect pools of either left or right plantaris muscles of *n* = 6 mice;(Chaillou et al*. *2013)); **d** hypoxia increases *Phgdh* expression after 6 h in mouse plantaris muscle (Gan et al*. *2017)

Collectively, these data suggest that the Hippo effector Yap in myoblasts not only promotes proliferation but might also drive the expression of *Phgdh* and *Psat1*. Based on these data, we sought to find out whether *Phgdh* is also regulated during different conditions of muscle perturbation in mice. As our reanalysis of published datasets shows in Fig. 3, *Phgdh* mRNA is increased 3 days after cardiotoxin injury (a), is highly expressed in dystrophic muscle (b), increases during days 3 to 7 after synergist ablation (c), and is induced by 6 h of hypoxia (d).

Together, these data suggest that *Phgdh* is highly expressed in muscles and tissues that contain high levels of proliferating myoblasts, a cell type where Yap is typically active (Judson et al*. *2012; Watt et al*. *2010).

**Fig. 4:**
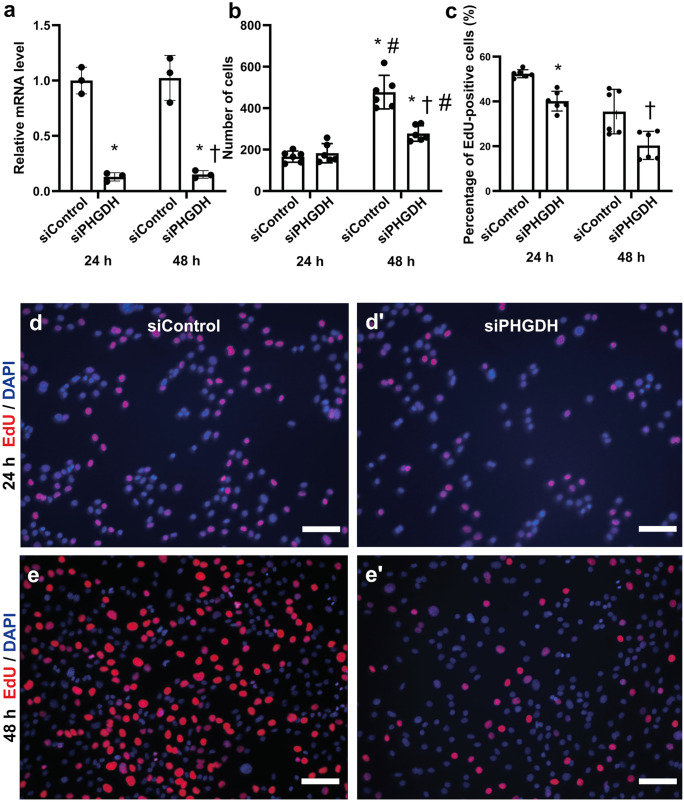
Effects of Phgdh on C2C12 myoblast proliferation. **a** siPHGDH reduces the relative mRNA level of *Phgdh* significantly after 24 and 48 h; **b** siPHGDH significantly reduces the number of cells after 48 h compared to siControl; **c** siPHGDH reduces the percentage of EdU-positive cells significantly after 24 and 48 h; **d–e′** EdU incorporation after 24- and 48-h treatment with siPHGDH. Scale bar represents 100 µm; *significantly different from 24 h siControl (*p* < 0.05); ^#^significantly different from 24-h siPHGDH (*p* < 0.05); ^†^significantly different from 48-h siControl (*p* < 0.05)

Next, we investigated whether *Phgdh* limits murine myoblast proliferation. To test this, we reduced Phgdh protein levels by siRNA and measured the proliferation rate by EdU assay. Using this strategy, we reduced *Phgdh* mRNA by 87% and 85% after 24- and 48-h transfection, respectively (Fig. 4a). When compared to control siRNA treatment, reduction of Phgdh by siRNA reduced proliferation by 42 ± 3% (*p* < 0.001) 48 h after transfection (Fig. 4b, d-e**′**). Transfection over 24 h did not significantly alter the number of total nuclei but reduced the number of EdU-positive cells by 23 ± 3% (*p* = 0.004) and 48 h reduced it by 48 ± 1% (*p* < 0.001) (Fig. 4c, d-e**′**).

**Fig. 5:**
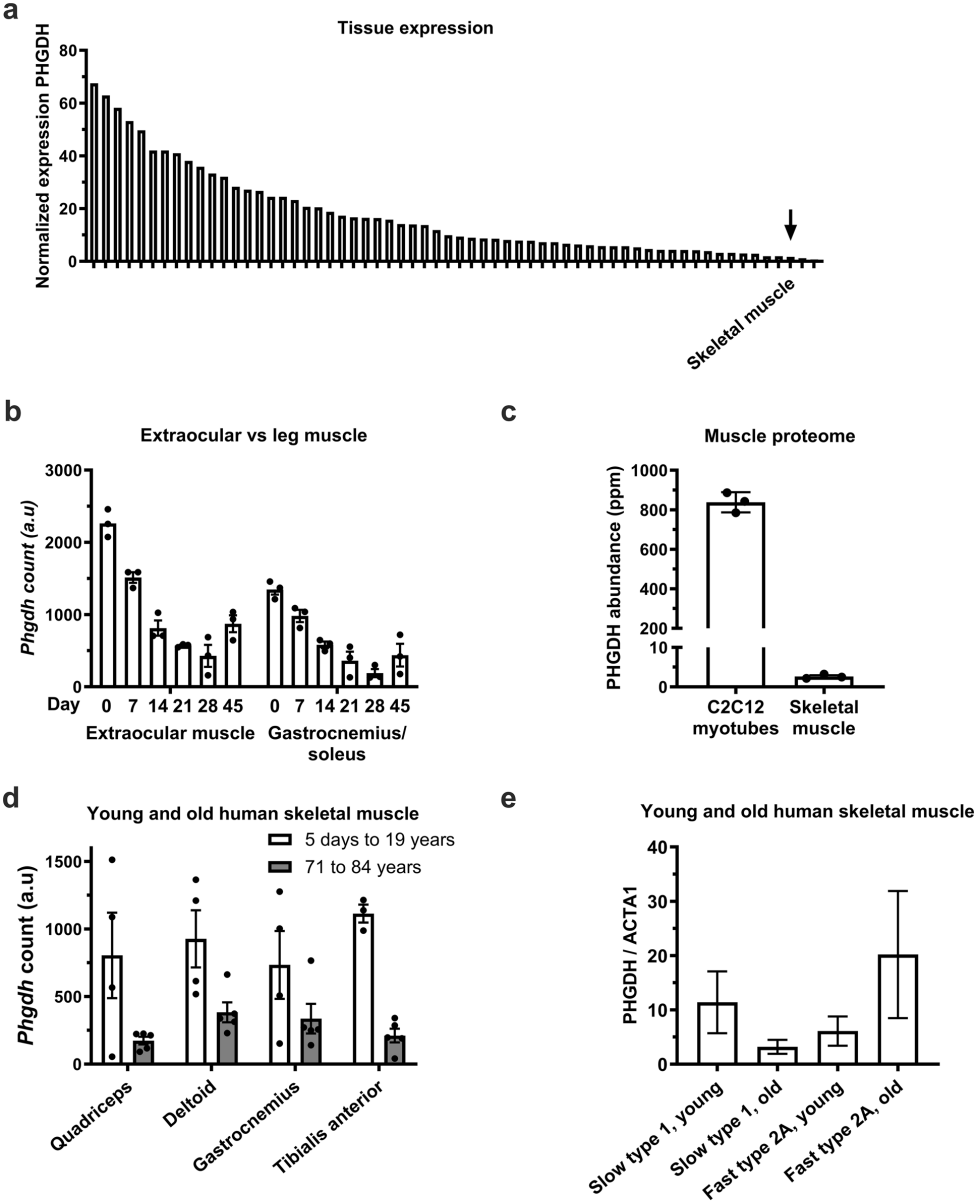
*Phgdh* expression across different human and rodent tissues and conditions. **a** Normalised PHGDH in skeletal muscle (arrow) in relation to different tissues in humans (expression data of all tissues are in Supplementary Table S1; www.proteinatlas.org (Uhlen et al*. *2015));** b** *Phgdh* expression is higher in fast extraocular muscles than in mixed gastrocnemius of Sprague–Dawley rats; x-axis represents age (Cheng et al*. *2004);** c** *PHGDH* expression is higher in C2C12 myotubes than in mouse triceps muscle (Deshmukh et al*. *2015); **d** *PHGDH* expression is higher in muscles of paediatric (5 days to 19 years) than in geriatric muscles (71 to 84 years) (Kang et al*. *2005); **e** normalised PHGDH protein is higher in slow type I muscle fibres of vastus lateralis in young humans (22–27 years) but lower in fast type 2A fibres compared to old humans (65–75 years) (Murgia et al*. *2017)

To examine the expression dynamics of these enzymes in vivo, we retrieved and reanalysed transcriptomic datasets that compared muscles and tissues with proliferating myoblasts where Yap is typically highly expressed and/or active with samples that contained fewer proliferating myoblasts (Fig. 5). We found that expression of *Phgdh* is generally low in skeletal muscle when compared to other tissues (Fig. 5a). Consistent between allotypes of muscles, *Phgdh* expression decreases in the first 28 days of development in rats but is higher in fast, extraocular muscle compared to mixed gastrocnemius muscle (Fig. 5b). We also show that PHGDH abundance is much higher in differentiating myotubes compared to skeletal muscle tissue (Fig. 5c) and is lower in skeletal muscles of older compared to younger humans (Fig. 5d). Single fibre proteomics of young and old men suggests that there is a trend that the normalised abundance of PHGDH is lower in vastus lateralis type I fibres of older people but higher in type IIa fibres when compared to younger men (Fig. 5e). Together, these data suggest that *Phgdh* may be regulated not only by age but possibly also in a fibre-specific manner.

## Discussion

The main finding of this study is that the Hippo effector Yap not only drives myoblast proliferation (Linch et al. 2014; Watt et al*. *2010) but is also associated with an increased expression of Phgdh. This is functionally relevant because a reduction of Phgdh reduces proliferation of myoblasts. Moreover, Phgdh is highly expressed in tissues and muscles with high levels of proliferating myoblasts, where Yap is typically active.

### Yap/Taz, Phgdh, and proliferation in skeletal muscle

Whilst Hippo signalling has been previously implicated in metabolic reprogramming of proliferating and cancer cells (Di Benedetto et al. 2021), a link between Yap and Phgdh expression has not been previously reported. We suggest that Yap drives Phgdh specifically in myoblasts. Yang et al. reported that Yap/Taz drive expression of the second enzyme of the serine biosynthesis pathway, *Psat1* (Yang et al. 2018). Moreover, muscles with high levels of activated satellite cells and proliferating myoblasts generally have high levels of Phgdh. This includes dystrophic muscles (Chemello et al*. *2020), regenerating mouse muscles (Lukjanenko et al*. *2013), and mouse muscles that hypertrophy because of synergist ablation (Chaillou et al*. *2013). Generally, these muscles and tissues also express high levels of the proliferation makers *Mki67* and *Pcna*.

### Yap/Taz-Phgdh in rhabdomyosarcoma

Rhabdomyosarcomas are childhood cancers. Alveolar rhabdomyosarcomas (ARMS) are typically driven by chimeric PAX3/PAX7-FOXO1 fusion genes. In contrast, embryonal rhabdomyosarcomas are driven by mutations of typical cancer genes (Shern et al. 2014). Previously, we found that YAP protein levels are higher in ERMS than ARMS and it was more nuclear (Tremblay et al*. *2014). Consistent with this, overexpression of constitutively active YAP1 S127A in activated satellite cells was sufficient to drive development of embryonal rhabdomyosarcomas in mice (Tremblay et al*. *2014). *Phgdh* and *Psat1* were 5.3-fold and 14.3-fold more expressed in YAP-driven rhabdomyosarcomas than in skeletal muscle, to which expression was compared (Tremblay et al*. *2014). This identifies the serine biosynthesis pathway as an active pathway in YAP-driven rhabdomyosarcomas. This adds YAP-driven embryonal rhabdomyosarcomas to cancers where the serine biosynthesis pathway and specifically Phgdh are highly expressed, often due to copy number gains including in breast cancer (Locasale et al*. *2011; Possemato et al*. *2011) and melanoma (Mullarky et al. 2011).

### Phgdh, proliferation, and mechanisms

We also found that lowering Phgdh by siRNA reduced the proliferation of C2C12 myoblasts, suggesting that Phgdh helps execute Yap-driven proliferation. The enzymatic function of Phgdh is to catalyse the first step of serine biosynthesis by transforming the glycolytic intermediate 3-phosphoglycerate into 3-phosphohydroxypyruvate. In theory, loss of Phgdh should have little consequence as it would turn serine into an essential amino acid that could be taken up in the diet. However, Phgdh-knockout mice are embryonal lethal (Yoshida et al*. *2004) suggesting that Phgdh has functions beyond serine synthesis. To find out whether and how Phgdh contributes to proliferation, Reid et al. (2018) inhibited Phgdh in HCT116 cells and report that pathways related to nucleotide synthesis were mainly affected (Reid et al. 2018). Proliferation assays further indicated that only supplementation with nucleosides rescued decreased proliferation upon Phgdh inhibition. This is in line with our finding that knockdown of Phgdh using siRNA decreases proliferation and, conversely, that Phgdh expression is higher in cells and tissues high in proliferating myoblasts. Gheller et al. (Gheller et al. 2021) showed that human muscle progenitor cells rely on extracellular serine and glycine for population expansion. De novo biosynthesis of serine was only detectable during serine/glycine restriction, but was only sufficient in the prevention of cell death. For us, this raises the question why Phgdh is upregulated in conditions where we expect a large number of proliferating cells and extracellular serine/glycine is not limiting. If de novo biosynthesis of serine/glycine is low, then it further supports the abovementioned idea that Phgdh limits proliferation in other ways than only serine biosynthesis.

### Phgdh-targeted therapies for muscle diseases?

Development of specific Phgdh inhibitors (McNamee et al. 2021; Mullarky et al. 2016; Pacold et al. 2016; Zhou et al. 2021) and ability to reduce dietary serine intake open up the possibility of targeting Phgdh and serine biosynthesis in pathologies where myogenic-related cells proliferate excessively. A prime target would be embryonal rhabdomyosarcoma where Phgdh expression is high (Tremblay et al*. *2014). Here, preclinical studies investigating the effectiveness of Phgdh inhibitors are warranted.

## Limitations

Whilst Phgdh is high in muscles and tissues with many proliferating myoblasts, this does not prove that Yap and Taz are drivers of Phgdh. Furthermore, we only demonstrate the proliferation-limiting effect of Phgdh in vitro. To prove that Phgdh is required for myoblast proliferation in vivo, an inducible, myoblast-specific mouse model is needed as global knockout of Phgdh is embryonal lethal (Yoshida et al. 2004).

## Summary and conclusion

In summary, high levels of the Hippo effectors Yap and Taz associate with an increased expression of *Phgdh*, whose knockdown limits proliferation of myoblasts. Consistent with this, Phgdh is highly expressed in muscles and tissues with proliferating myoblasts when compared to controls tissues with few proliferating myoblasts. The advent of Phgdh inhibitors and ability to manipulate serine ingestion in the diet offer therapeutic tools to try to normalise Phgdh levels in diseases.

### Supplementary Information

Below is the link to the electronic supplementary material.

Supplementary file1 (DOCX 15 KB)

## Data Availability

The data supporting our findings are available from the corresponding author upon reasonable request.
