# Supplementary material for: Yap/Taz activity is associated with increased expression of phosphoglycerate dehydrogenase that supports myoblast proliferation
Source: Cell Tissue Res. 2024 Jan 6;395(3):271–83. doi: 10.1007/s00441-023-03851-w (PMC10904560; doi:10.1007/s00441-023-03851-w)
Supplement: Supplementary file 1 — Supplementary file1 (DOCX 15 KB) [file 441_2023_3851_MOESM1_ESM.docx]

**Yap/Taz activity is associated with increased expression of phosphoglycerate dehydrogenase which limits myoblast proliferation**

Marius Meinhold^1*^, Sander Verbrugge^1^, Andi Shi^2,3^, Martin Schönfelder^1^, Lore Becker^5^, Richard T. Jaspers^2^, Peter S. Zammit^4^ & Henning Wackerhage^1^

^1^Technische Universität München, Associate Professorship of Exercise Biology, Connollystrasse 32, 80809 Munich, Germany

^2^Laboratory for Myology, Department of Human Movement Sciences, Faculty of Behavioural and Movement Sciences, Vrije Universiteit Amsterdam, Amsterdam Movement Sciences, De Boelelaan 1108, 1081 HZ Amsterdam, The Netherlands

^3^Department of Prosthodontics, Affiliated Stomatology Hospital of Guangzhou Medical University, Guangdong Engineering Research Center of Oral Restoration and Reconstruction, Guangzhou Key Laboratory of Basic and Applied Research of Oral Regenerative Medicine, Guangzhou, China

^4^ King's College London, Randall Centre for Cell and Molecular Biophysics, New Hunt's House, Guy's Campus, London SE1 1UL, UK

^4^Helmholtz Zentrum München, German Research Center for Environmental Health, Intitute of Experimental Genetics, German Mouse Clinic, Ingolstädter Landstrasse 1, 85764 Neuherberg, Germany

*Correspondence to m.meinhold@tum.de

**Supplementary Table S1. Normalised tissue expression of PHGDH (proteinatlas.org)**

| **Organ** | **Normalised expression of PHGDH** |
| --- | --- |
| parathyroid gland | 67,5 |
| salivary gland | 62,9 |
| pancreas | 58,2 |
| liver | 53,2 |
| corpus callosum | 49,7 |
| midbrain | 42 |
| substantia nigra | 42 |
| epididymis | 41 |
| thalamus | 38,1 |
| skin | 35,9 |
| amygdala | 33,3 |
| basal ganglia | 32,1 |
| spinal cord | 28,3 |
| cerebral cortex | 27,2 |
| hippocampal formation | 26,7 |
| fallopian tube | 24,5 |
| prostate | 24,5 |
| pons and medulla | 23,3 |
| olfactory region | 20,7 |
| hypothalamus | 20,5 |
| adipose tissue | 18,8 |
| ovary | 17,3 |
| esophagus | 16,7 |
| T-cells | 16,5 |
| seminal vesicle | 16,4 |
| thyroid gland | 15,8 |
| vagina | 14,1 |
| kidney | 14 |
| breast | 13,8 |
| cervix, uterine | 11,9 |
| thymus | 9,9 |
| ductus deferens | 9,4 |
| stomach | 9 |
| retina | 8,7 |
| testis | 8,6 |
| smooth muscle | 8,1 |
| urinary bladder | 7,9 |
| cerebellum | 7,8 |
| tongue | 7,3 |
| tonsil | 7,3 |
| B-cells | 6,7 |
| placenta | 6,4 |
| endometrium | 6,1 |
| colon | 5,8 |
| lymph node | 5,7 |
| pituitary gland | 5,7 |
| heart muscle | 5,32 |
| rectum | 4,7 |
| appendix | 4,4 |
| bone marrow | 4,4 |
| lung | 4,3 |
| duodenum | 4,2 |
| gallbladder | 3,9 |
| dendritic cells | 3,2 |
| total PBMC | 3,2 |
| small intestine | 3 |
| granulocytes | 2,9 |
| adrenal gland | 1,9 |
| spleen | 1,9 |
| skeletal muscle | 1,7 |
| NK-cells | 1,2 |
| monocytes | 0,7 |
